# Parents’ Perspectives on Their Relationship With Their Adolescent Children With Internet Addiction: Survey Study

**DOI:** 10.2196/35466

**Published:** 2022-10-05

**Authors:** Hideki Horita, Yoichi Seki, Eiji Shimizu

**Affiliations:** 1 Department of Cognitive Behavioral Physiology Graduate School of Medicine Chiba University Chiba Japan; 2 International University of Health and Welfare Narita Japan; 3 Cognitive Behavioral Therapy Center Chiba University Hospital Chiba Japan; 4 Research Center for Child Mental Development Chiba University Chiba Japan

**Keywords:** internet addiction, mental health, parent-child relationship

## Abstract

**Background:**

Parents of adolescents with internet addiction are confronted with their children’s internet problems on a daily basis. Parents may notice that adolescents with addiction may also have emotional and behavioral problems, including impulsivity and violence. Parenting styles have been found to be related to internet addiction.

**Objective:**

The purpose of this study is to investigate parents’ perspectives on their parenting style, relationship with their child, and the degree of internet addiction and emotional and behavioral problems of their child.

**Methods:**

A web survey was conducted with 600 parents of children between the ages of 12 and 17 years, from October 14 to 18, 2021, across Japan. Respondents were recruited by an internet research company and were asked to complete an anonymous online questionnaire. The survey was divided into two groups: 300 parents who answered “yes” to the question “Do you think your child is dependent on the internet?” and 300 parents who answered “no” to that question. Questionnaires were collected until each group had 300 participants. The questionnaire included (1) the Parent-Child Internet Addiction Test (PCIAT), (2) the daily time spent using the internet, (3) the Strengths and Difficulties Questionnaire (SDQ), (4) the Parenting Style and Dimensions Questionnaire (PSDQ), and (5) the Relationship Questionnaire (RQ) measuring self-report attachment style prototypes.

**Results:**

Mean scores of the PCIAT and the daily time spent using the internet for the group with probable internet addiction were significantly higher than those of the group without probable internet addiction (50%; *P*<.001). The total difficulties score from the SDQ for the group with probable internet addiction (mean 10.87, SD 5.9) was significantly higher than that for the group without probable internet addiction (mean 8.23, SD 5.64; *P*<.001). The mean score for authoritarian parenting from the PSDQ for the group with probable internet addiction (mean 2.1, SD 0.58) was significantly higher than that for the group without probable internet addiction (mean 2.1, SD 0.58; *P*<.001). Regarding the RQ, there were no significant differences between the two groups.

**Conclusions:**

Our findings suggest that parents who think their child is addicted to the internet may recognize emotional and behavioral problems of the child and have an authoritarian parenting style.

## Introduction

The internet is a highly convenient tool for the instantaneous and comprehensive exchange of large amounts of information with the world. It is no exaggeration to say that our lives are directly or indirectly supported by the internet, and it has enriched our lives through information accessibility, entertainment, communication, and trading. Over the past decade, internet use has increased dramatically and has become an integral part of everyday life. It has become especially central among adolescents and emerging adults, for whom technological literacy is important for both work and play. Recently, however, the negative aspects of the internet have been attracting attention, and in addition to fraud, crime, bullying, and wastage of time via the internet, the problem of internet dependence, the subject of this study, has been highlighted [1-4].

It was not until 1990 that reports of internet dependence began to appear sporadically. Overuse of the internet causes serious problems, such as poor grades, withdrawal to one’s room, disordered eating habits, and lack of sleep. On the mental side, it causes depression, aggression, worsening of general mental symptoms, and a decline in self-esteem, which is undesirable for an individual’s career path and social support [5]. The line between internet use and problematic internet use has been significantly overstepped. The concept of “addiction” has raised interest in the study of the internet. Problematic internet use comprises an important area of research as its negative effects have been found to affect daily functioning, interpersonal relationships, and emotional well-being [6-8]. In addition, its symptoms resemble those of substance-related addictions, including unpredictable behaviors and moods [9,10].

Due to this trend, the diagnostic criteria for internet gaming disorder (IGD) were included in the Diagnostic and Statistical Manual of Mental Disorders, Fifth Edition (DSM-5) in 2013 [11]. In addition, the International Classification of Diseases, 11th Revision, published by the World Health Organization in June 2018, also included diagnostic criteria for gaming disorder [12]. Pan et al [13] conducted a systematic review and meta-analysis of 113 studies that included 693,306 subjects. The 133 effect sizes included 53,184 subjects, and the authors reported that the weighted average prevalence for generalized internet addiction and IGD were 7.02 % and 2.47 %, respectively. A review of psychological intervention studies for internet addiction found the following interventions: cognitive behavior therapy, family therapy, reality training, cognitive bias modification, craving behavioral intervention, and integration of psychological treatments [14].

In recent years, various studies have been conducted on adolescents with internet addiction. It has been found that among junior high school students both attention-deficit/hyperactivity disorder (ADHD) and autism spectrum disorder (ASD), caused by developmental disabilities, are related to the risk of internet dependence.

We believe that parents’ perspectives on their child’s internet addiction are important because parenting a child with internet addiction as well as ASD, ADHD, or both can be a challenging and difficult experience. Simply scolding or punishing their children’s internet addiction is a bad form of communication by parents and might exacerbate the internet addiction, leading to a vicious cycle. In such cases, we believe that helping parents with their children suffering from internet addiction through cognitive behavioral therapy [15,16], especially Community Reinforcement Approach and Family Training (CRAFT) [17,18], will be useful. Psychotherapy for the parent may improve the relationship between parent and child and stop the vicious cycle of internet addiction.

Based on this hypothesis, we are conducting a pilot randomized controlled trial of videoconference-based cognitive behavioral therapy for parents with children suffering from internet addiction between the ages of 12 and 20 years, separately from this study, which was approved by the Ethics Review Committee of Chiba University Hospital in 2018 (UMIN 000032483).

Direct parental factors, such as lack of affection from parents, increase children’s online dependence. While a good parent-child relationship is negatively associated with online dependence, particularly among adolescents, there are reports that parents’ discord is associated with increased online dependence among children [19,20].

The purpose of this quantitative study was to compare parents’ perspectives on the degree of their child’s internet addiction and emotional and behavioral problems, their parenting style, and the parent-child relationship between parents with children afflicted with internet addiction and those without, using an anonymous web-based survey across Japan.

## Methods

### Participants

We used an online research agency (Cross Marketing Inc, Tokyo) to oversee the web-based survey from October 14 to 18, 2021, across Japan. After understanding the purpose of the study and voluntarily agreeing to participate, 600 participants from Japan were recruited through the online research provider.

The participants were parents with children between the ages of 12 and 17 years, and they were asked to fill out an anonymous online questionnaire. Parents were instructed to complete the survey about only 1 child with internet addiction, no matter how many children they had.

We asked the parents, “Please think of your child who is addicted to the internet” and “Please tell us the birth order of that child.”

The survey was divided into two groups: 300 parents who answered “yes” to the question “Do you think your child is dependent on the internet?” and 300 parents who answered “no” to that question. Questionnaires were collected until the number of parents in each group reached 300.

### Items for Observation, Examination, Survey, and Reporting

#### Overview

Candidate respondents received brief text-based information about the study, including the purpose of the study, and informed consent was obtained. The survey consisted of 2 parts. The first part asked for general information about the respondents (ie, age, gender, area of residence, and employment status of the parents, as well as age, gender, birth order, and hours of internet use per day of their children).

The second part of the survey asked respondents to selectively answer the 4 questionnaire items described in the following sections.

#### Parent-Child Internet Addiction Test

The items of the questionnaire pertaining to children’s internet addiction from the parents’ points of view were adapted from the Parent-Child Internet Addiction Test (PCIAT) [21-23], a 20-item inventory adapted from the Internet Addiction Test (IAT) developed by Young [24]. Items were rated on a 5-point Likert scale, ranging from 1 (not at all) to 5 (frequently), to indicate the degree to which internet use affected daily life, family relationships, social life, personal health, and state of mind. The minimum score was 20 and the maximum score was 100, with higher scores indicating greater problems caused by internet use. Young defines a score of 20 to 49 as an average user who has control over their use of the internet, a score of 50 to 79 as a dependent user who has occasional or frequent problems with their use of the internet, and a score of 80 to 100 as a dependent user who has major problems with their use of the internet.

#### Strengths and Difficulties Questionnaire

The Strengths and Difficulties Questionnaire (SDQ), developed by Goodman [25,26], is a comprehensive measure of children’s adjustment and mental health status. It is a highly reliable screening method for assessing positive and negative aspects of children’s behavior [27].

The SDQ consists of 25 items, with 5 subscales (ie, emotional symptoms, conduct problems, hyperactivity-inattention, peer problems, and prosocial behavior) and 5 items within each subscale. Each question was answered by selecting from 3 options: “yes” (2 points), “fairly true” (1 point), and “no” (0 points). The total score for each subscale was calculated, and the total difficulties score (TDS) was calculated from the total score of 4 of the 5 subscales: the prosocial behavior subscale was excluded.

In addition, by setting a cutoff point, the need for support in that area was classified into 3 categories: normal range, borderline range, and clinical range.

#### Parenting Style and Dimensions Questionnaire

The Parenting Style and Dimensions Questionnaire (PSDQ) by Robinson et al [28], which consists of subscales based on Baumrind’s [29] classification of authoritative, authoritarian, and permissive parents, was used. It measures various characteristics of parents and children [30,31] and is an excellent scale for measuring parents’ nurturing attitudes.

#### Self-Report Attachment Style Prototypes: Relationship Questionnaire

The Relationship Questionnaire (RQ), which measures 4 categories of attachment style, was used to measure the attachment styles of parents and children. Bartholomew et al’s [32-34] RQ consists of a statement describing the characteristics of 4 attachment styles in relation to the “general other.” Subjects were first asked to rate the degree to which each of the 4 sentences introduced as “types of feelings toward people” matched their own on a 7-point scale, ranging from 1 (not at all) to 7 (very much). Next, they were asked to choose 1 of the 4 styles that they thought was the most applicable to them. In the analysis, the attachment style chosen at the end was considered the subject’s attachment style.

### Statistical Analysis

A descriptive analysis (ie, numbers, frequencies, percentages, means, and SDs) of the 600 respondents was conducted. The responses of the 300 respondents in the “yes” group and the 300 respondents in the “no” group were compared for differences in items using a *t* test. Frequencies of gender, marital status, and birth order were analyzed using the chi-square test or the Fisher exact test. For the characteristics of the participants, *P* values were considered by applying a 2-tailed significance level of less than .05. For the SDQ, the PSDQ, and the RQ, we used the Bonferroni correction and set the *P* value threshold of .05/19=.0026 in order to avoid increasing the risk of a type I error by multiple comparisons. All data were analyzed with SPSS (version 22.0; IBM Corp).

### Ethics Approval

This study was approved by the Ethical Review Committee of the Graduate School of Medicine, Chiba University, in September 2021 (M10095).

## Results

### Overview

The characteristics of the participants are shown in Table 1. The mean age of the parents was 49.24 (SD 5.67) years in the “yes” group and 49.07 (SD 5.06) years in the “no” group, with no significant difference between the two groups. Regarding marital status, about 95.1% (571/600) of the respondents in both groups were married; there was no significant difference between groups. There were significant differences in gender between the two groups. Female participants in the “yes” group constituted 45.0% (135/300) of the sample, whereas in the “no” group they constituted 36.0% (108/300) of the sample.

The average age of the participants’ children was 15.01 (SD 1.59) years in the “yes” group and 14.95 (SD 1.58) years in the “no” group, with no significant difference between the groups. In terms of birth order, 58.7% (352/600) of the adolescents were the first child, 31.5% (189/600) were the second child, 8.0% (48/600) were the third child, and 1.8% (11/600) were in a different birth order position, with no significant difference (Table 1).

The total PCIAT score for the group that answered “yes” (mean 55.41, SD 15.78) was significantly higher than that for the group that answered “no” (mean 35.55, SD 11.64). As for the daily time spent on the internet, the children in the group that answered “yes” spent a mean of 4.0 (SD 2.06) hours on the internet, and those in the group that answered “no” spent a mean of 1.7 (SD 1.06) hours on the internet, and there was a statistically significant difference between the two groups (*P*<.001).

**Table 1:** Characteristics of the participants.

Characteristics				Parents who thought their child was addicted to the internet (n=300)	Parents who did not think their child was addicted to the internet (n=300)	*P* value	
**Parent**							
	**Age (years)**						
		Mean (SD)	49.24 (5.67)		49.07 (5.06)	.69	
		Range	35-65		33-64	N/A^a^	
	**Gender, n (%)**						.03^b^
		Male	165 (55.0)		192 (64.0)		
		Female	135 (45.0)		108 (36.0)		
	**Marital status, n (%)**						>.99
		Married	284 (94.7)		287 (95.7)		
		Single	16 (5.3)		13 (4.3)		
**Adolescent**							
	**Age (years)**						
		Mean (SD)	15.01 (1.59)		14.95 (1.58)	.61	
		Range	12-17		12-17	N/A	
	**Gender, n (%)**						>.99
		Male	165 (55.0)		165 (55.0)		
		Female	134 (44.7)		134 (44.7)		
		No answer	1 (0.3)		1 (0.3)		
	**Birth order, n (%)**						>.99
		1st child	180 (60.0)		172 (57.3)		
		2nd child	93 (31.0)		96 (32.0)		
		3rd child	23 (7.7)		25 (8.3)		
		Other	4 (1.3)		7 (2.3)		
	**PCIAT^c^ total score**						
		Mean (SD)	55.41 (15.78)		35.55 (11.64)	<.001	
		Range	21-98		21-74	N/A	
	**Daily time spent using the internet (hours)**						
		Mean (SD)	4.0 (2.06)		1.7 (1.06)	<.001	
		Range	0-17		0-7	N/A	

^a^*P* values were not calculated for range values.

^b^*P* values for a group are reported in the main row of the group.

^c^PCIAT: Parent-Child Internet Addiction Test; scores ranged from 20 to 100, with higher scores indicating greater problems caused by internet use.

### Comparison of SDQ, PSDQ, and RQ Values From Both Groups

The results of the SDQ, the PSDQ, and the RQ are shown in Table 2. In the SDQ, the mean TDS score for the group that answered “yes” was significantly higher than for the group that answered “no” (*P*<.001). Regarding the subscale items, mean scores for emotional symptoms, conduct problems, and hyperactivity-inattention for the “yes” group were significantly higher than those for the “no” group (*P*<.001). There were no significant differences between groups regarding peer problems (*P*<.049) and prosocial behavior (*P*<.13).

Regarding the PSDQ, the mean score for authoritarian parenting of the “yes” group was significantly higher than that of the “no” group (*P*<.001). There were no significant differences between authoritative parenting and permissive parenting. Regarding the RQ, there was no statistically significant difference between the “yes” and “no” groups of parents and children on the whole, whether they had secure, dismissive, preoccupied, or fearful relationships.

**Table 2:** Comparison of Strengths and Difficulties Questionnaire (SDQ), Parenting Style and Dimensions Questionnaire (PSDQ), and Relationship Questionnaire (RQ) results between groups.

Scales and subscales				Parents who thought their child was addicted to the internet (n=300), mean (SD)		Parents who did not think their child was addicted to the internet (n=300), mean (SD)		*P* value
**SDQ^a^ score (25 items, including all 5 subscales)**								
	Total difficulties score (20 items, excluding prosocial behavior subscale)			10.87 (5.91)		8.23 (5.64)		<.001
	**Subscales (5 items each)**							
		Emotional symptoms	2.04 (2.18)		1.41 (1.83)		<.001	
		Conduct problems	2.26 (1.75)		1.51 (1.46)		<.001	
		Hyperactivity-inattention	3.70 (2.17)		2.73 (2.12)		<.001	
		Peer problem	2.87 (1.84)		2.58 (1.76)		.049	
		Prosocial behavior	4.81 (2.44)		5.11 (2.32)		.13	
**PSDQ^a^ score (62 items)**								
	Authoritative subscale (27 items)			3.11 (0.61)		3.14 (0.67)		.65
	Authoritarian subscale (20 items)			2.27 (0.61)		2.10 (0.58)		.001
	Permissive subscale (15 items)			2.37 (0.44)		2.28 (0.46)		.02
**RQ^a^ score**								
	**Parent**							
		Secure	3.83 (1.46)		3.85 (1.29)		.88	
		Dismissing	3.70 (1.38)		3.90 (1.36)		.07	
		Preoccupied	3.85 (1.31)		3.76 (1.35)		.41	
		Fearful	3.73 (1.47)		3.78 (1.41)		.71	
	**Adolescent**							
		Secure	4.20 (1.32)		4.24 (1.22)		.70	
		Dismissing	3.70 (1.26)		3.60 (1.13)		.29	
		Preoccupied	3.97 (1.15)		3.87 (1.1)		.31	
		Fearful	3.52 (1.31)		3.27 (1.19)		.01	

^a^For the SDQ, the PSDQ, and the RQ, we used the Bonferroni correction and set the *P* value threshold of .05/19=.0026 in order to avoid increasing the risk of a type I error by multiple comparisons.

### Comparison of High Internet Users Versus Low Internet Users

From the 300 parents who answered “yes,” we extracted those who scored 50 or higher on the PCIAT (190/300, 63.3%) to examine users who experienced occasional or frequent problems due to internet use. From the 300 parents who answered “no,” 86.0% (258/300) had a PCIAT score of less than 50. The 2 sets were compared to each other. The results of the SDQ, the PSDQ, and the RQ are shown in Table 3.

The SDQ showed statistically significant differences in the TDS and in the subscales of emotional symptoms, conduct problems, hyperactivity-inattention, peer problems, and prosocial behavior (all *P*s<.001). The PSDQ showed a significant difference between authoritarian and permissive parents (*P*<.001) but not authoritative parents (authoritative subscale: *P*=.24; authoritarian subscale: *P*<.001; permissive subscale: *P*<.001).

Regarding the RQ, no statistically significant differences were found for parents under any of the items. Conversely, the children showed a significant difference only in the fearful type (*P*<.001).

We regrouped participants with a PCIAT cutoff value of 50, ignoring whether parents thought their child was addicted to the internet or not, conducted the analysis, and made a new table (Table 4).

The results showed that the group with a PCIAT score of 50 or higher (n=232) had a PCIAT mean score of 63.58 (SD 10.49). The group with a PCIAT score lower than 50 (n=368) had a PCIAT mean score of 34.07 (SD 8.19). The results of the SDQ, the PSDQ, and the RQ were compared between the two groups and were similar to those in Table 3.

**Table 3:** Comparison of parents who thought their child was addicted to the internet (Parent-Child Internet Addiction Test [PCIAT] score ≥50) and those who did not (PCIAT score <50).

Scales and subscales						Parents who thought their child was addicted to the internet (n=190), mean (SD)			Parents who did not think their child was addicted to the internet (n=258), mean (SD)			*P* value
PCIAT total score						64.91 (10.91)			31.96 (7.8)			<.001
**SDQ^a,b^ score (25 items, including all 5 subscales) **												
	Total difficulties score (20 items, excluding prosocial behavior subscale)				12.55 (5.65)			7.41 (4.96)			<.001	
	**Subscales (5 items each)**											
			Emotional symptoms		2.44 (2.25)			1.17 (1.6)			<.001	
			Conduct problems		2.61 (1.81)			1.3 (1.25)			<.001	
			Hyperactivity-inattention		4.33 (2.09)			2.52 (2.02)			<.001	
			Peer problem		3.17 (1.88)			2.42 (1.68)			<.001	
			Prosocial behavior		4.36 (2.33)			5.15 (2.35)			<.001	
**PSDQ^b,c^ score (62 items)**												
	Authoritative subscale (27 items)				3.09 (0.58)			3.16 (0.68)			.24	
	Authoritarian subscale (20 items)				2.39 (0.58)			2.04 (0.56)			<.001	
	Permissive subscale (15 items)				2.43 (0.4)			2.22 (0.45)			<.001	
**RQ^b,d^ score**												
	**Parent**											
		Secure		3.75 (1.43)			3.86 (1.33)			.38		
		Dismissing		3.59 (1.35)			3.88 (1.4)			.03		
		Preoccupied		3.95 (1.28)			3.73 (1.38)			.09		
		Fearful		3.79 (1.48)			3.74 (1.47)			.71		
	**Adolescent**											
		Secure		4.08 (1.32)			4.26 (1.23)			.14		
		Dismissing		3.74 (1.27)			3.56 (1.14)			.12		
		Preoccupied		4.06 (1.21)			3.84 (1.13)			.046		
		Fearful		3.66 (1.34)			3.2 (1.22)			<.001		

^a^SDQ: Strengths and Difficulties Questionnaire.

^b^For the SDQ, the PSDQ, and the RQ, we used the Bonferroni correction and set the *P* value threshold of .05/19=.0026 in order to avoid increasing the risk of a type I error by multiple comparisons.

^c^PSDQ: Parenting Style and Dimensions Questionnaire.

^d^RQ: Relationship Questionnaire.

**Table 4:** Comparison of 600 subjects classified according to Parent-Child Internet Addiction Test (PCIAT) cutoff values.

Scales and subscales					PCIAT score ≥50, (n=232)		PCIAT score <50 (n=368)		*P* value
PCIAT total score, mean (SD)					63.58 (10.49)		34.07 (8.19)		<.001
Parents who thought their child was addicted to the internet, n (%)					190 (81.9)		110 (29.9)		N/A^a^
Parents who did not think their child was addicted to the internet, n (%)					42 (18.1)		258 (70.1)		N/A
**SDQ^b,c^ score (25 items, including all 5 subscales), mean (SD)**									
	Total difficulties score (20 items, excluding prosocial behavior subscale)			12.67 (5.88)		7.58 (5.03)		<.001	
	**Subscales (5 items each)**								
			Emotional symptoms	2.51 (2.28)		1.23 (1.68)		<.001	
			Conduct problems	2.64 (1.82)		1.4 (1.33)		<.001	
			Hyperactivity-inattention	4.27 (2.1)		2.55 (1.97)		<.001	
			Peer problem	3.24 (1.88)		2.39 (1.67)		<.001	
			Prosocial behavior	4.45 (2.29)		5.29 (2.38)		<.001	
**PSDQ^c,d^ score (62 items), mean (SD)**									
	Authoritative subscale (27 items)			3.08 (0.58)		3.16 (0.67)		.12	
	Authoritarian subscale (20 items)			2.41 (0.57)		2.05 (0.58)		<.001	
	Permissive subscale (15 items)			2.47 (0.41)		2.23 (0.46)		<.001	
**RQ^c,e^ score, mean (SD)**									
	**Parent**								
		Secure		3.75 (1.36)		3.9 (1.38)		.19	
		Dismissing		3.68 (1.32)		3.88 (1.4)		.07	
		Preoccupied		3.95 (1.25)		3.72 (1.37)		.04	
		Fearful		3.83 (1.41)		3.71 (1.46)		.30	
	**Adolescent**								
		Secure		4.09 (1.29)		4.31 (1.25)		.04	
		Dismissing		3.76 (1.23)		3.58 (1.17)		.09	
		Preoccupied		4.07 (1.15)		3.83 (1.1)		.01	

^a^N/A: not applicable; a *P* value was not calculated for this item.

^b^SDQ: Strengths and Difficulties Questionnaire.

^c^For the SDQ, the PSDQ, and the RQ, we used the Bonferroni correction and set the *P* value threshold of .05/19=.0026 in order to avoid increasing the risk of a type I error by multiple comparisons.

^d^PSDQ: Parenting Style and Dimensions Questionnaire.

^e^RQ: Relationship Questionnaire.

## Discussion

### Principal Findings

In this study, we administered a questionnaire to investigate the relationship between parenting styles and adolescents’ internet addiction and mental health problems. In recent years, various studies on adolescents have suggested that internet dependence is associated with developmental disorders; in addition, both ADHD and ASD have been found to be associated with the risk of internet dependence. Cakmak and Gul [35] reported that weekly internet usage among children with ADHD aged 12 to 16 years was higher than among children in the control group. Kawabe et al [36] reported that 25 out of 55 participants with ASD were classified as having internet addiction based on results from the IAT.

In this study, we did not take into account the diagnosis of ADHD, ASD, or both, but we did measure SDQ scores and found that the TDS of the SDQ in the group with internet addiction was significantly higher than that in the group without addiction. Baer et al [37] reported that the Computer/Gaming-station Addiction Scale score significantly correlated with the total SDQ score. Akdeniz et al [38] reported that the TDS of the SDQ was higher in the group with internet addition compared to that of the group without internet addiction. Our findings were consistent with previous studies.

Previous research on the parent-child relationship between internet-dependent adolescents and their parents has largely been conducted from the perspective of the adolescents [39-41]. In this study, the perspective of the parents was the focus, and we investigated the parenting styles of those parents with internet-dependent adolescents. In a previous study from the parents’ perspective, Dogan et al [42] investigated the perceptions of internet addiction and parenting styles among adolescents studying in secondary schools between the ages of 14 and 19 years. They used the Parental Attitude Scale by Kuzgun and Eldeleklioğlu [43] to measure parental attitude, and the results showed a negative relationship between internet addiction and a democratic parenting style. Results of that study also showed a negative relationship between a protective-demanding parenting style and an authoritarian parenting style, which was found to have a significant positive relationship with internet addiction. This study used the PSDQ, a parenting style scale created by Robinson et al [28]; the results from Robinson et al’s study were consistent with the findings from our study, showing that parents in the group with internet-dependent children were found to have significantly higher authoritarian parenting tendencies than parents in the group with children who were not dependent on the internet.

Dogan et al [42] also found that a protective-demanding parenting style was a strong predictor of internet dependence, followed by an authoritarian parenting style. Although the 3 subscales of the PSDQ in this study and the 3 subscales of Kuzgun and Eldeleklioğlu’s Parental Attitude Scale in the study by Dogan et al [42] are not comparable, the findings with regard to the relationship of authoritarian parenting style with internet addiction may be common.

Using structural equation modeling analyses of the data from 266 adolescents, Trumello et al [44] suggested that adolescents’ mental health problems measured by the SDQ are an important mediator between parental care and youths’ internet addiction. Our findings were in accordance with their report.

The research implications of this study are that parents who have children with internet addiction may be more aware of their children’s emotional and behavioral problems, and their parenting style is more authoritarian.

Clinicians may encourage parents to stop their authoritarian parenting style, to learn good communication skills, and to reward their children when they choose desirable behaviors. They may also encourage parents to engage children in treatment for internet abuse and emotional and behavioral problems using cognitive behavioral therapy, especially the CRAFT intervention, at the end of their discussion as their recommendation.

### Limitations

As we suggested, although the online survey conducted in this study provided valuable information, it has several limitations. The first limitation was that we did not use the random sampling method. Originally, it would have been ideal to conduct random sampling, in which the probability of being selected for the sample would be equal for all individuals. In the future, an online survey using the random sampling method should be conducted.

The second limitation was that the children in this study were not diagnosed according to the DSM-5 diagnostic criteria for IGD. A structured diagnostic interview based on the online survey about internet addiction will be needed.

The third limitation was that no data were collected from the children in this survey. The parents’ evaluations of their children were based on their assumptions. Considering that there are differences in the understanding of internet addiction between parents and children, future research should focus on collecting data from both parents and children.

### Conclusions

ADHD and ASD are known to be related to the risk of internet addiction. Our findings suggest that parents who think their child is addicted to the internet may recognize emotional and behavioral problems in the child measured by the SDQ. In addition, parents with children who suffer from internet addiction may have an authoritarian parenting style. Clinicians may encourage parents to learn good communication skills instead of an authoritarian parenting style.

In the future, studies should conduct additional research on internet addiction in children and their families. Cross-sectional and longitudinal research on families, especially parents, is also needed.

## Abbreviations

ADHD: attention-deficit/hyperactivity disorder
ASD: autism spectrum disorder
CRAFT: Community Reinforcement Approach and Family Training
DSM-5: Diagnostic and Statistical Manual of Mental Disorders, Fifth Edition
IAT: Internet Addiction Test
IGD: internet gaming disorder
PCIAT: Parent-Child Internet Addiction Test
PSDQ: Parenting Style and Dimensions Questionnaire
RQ: Relationship Questionnaire
SDQ: Strengths and Difficulties Questionnaire
TDS: total difficulties score
